# Supplementary material for: Genomic, Molecular, and Phenotypic Characterization of Arthrobacter sp. OVS8, an Endophytic Bacterium Isolated from and Contributing to the Bioactive Compound Content of the Essential Oil of the Medicinal Plant Origanum vulgare L
Source: Int J Mol Sci. 2023 Mar 2;24(5):4845. doi: 10.3390/ijms24054845 (PMC10002853; doi:10.3390/ijms24054845)

Supplementary Material

Table S1. Average Nucleotide Identity (ANI) analysis results. Only the first 10 reference genomes showing the higher ANI values were here reported.

Reference genome	Species	ANI value (%)	Bidirectional Fragment Mappings	Total Query Fragments
GCF_019977335_1_ASM1997733v1	Arthrobacter sp. NicSoilB4	89.5916	1102	1391
GCF_001750145_1_ASM175014v1	Arthrobacter sp. U41	88.0.188	1011	1391
GCF_004798705_1_ASM479870v1	Arthrobacter sp. PAMC25564	84.7887	872	1391
GCF_007858535_1_ASM785853v1	Arthrobacter sp. UKPF54-2	84.7483	890	1391
GCF_947090775_1_MM222	Pseudarthrobacter sp. MM222	84.6723	961	1391
GCF_019977355_1_ASM1997735v1	Arthrobacter sp. NicSoilB8	84.3877	969	1391
GCF_005937985_2_ASM593798v2	Arthrobacter sp. KBS0702	84.3611	875	1391
GCF_005280255_1_ASM528025v1	Arthrobacter sp. 24S4-2	82.8444	841	1391
GCF_000196235_1_ASM19623v1	Arthrobacter sp. FB24	82.6295	858	1391
GCF_019443425_1_ASM1944342v1	Arthrobacter sp. PAMC25284	82.4347	782	1391

Table S2. Results of the Type Strain Genome Server (TYGS) species identification routine.

Query strain	Conclusion
Arthrobacter sp. OVS8	Potential new species

Table S3. Pairwise comparisons of *Arthrobacter* sp. OVS8 genome vs. type strain genomes obtained with the TYGS platform. The following table contains the pairwise digital DNA-DNA hybridization (dDDH) values between *Arthrobacter* sp. OVS8 genome and the selected type strain genomes. The dDDH values are provided along with their confidence intervals (C.I.) for the three different Genome Blast Distance Phylogeny (GBDP) formulas: formula d0, length of all high-scoring segment pair (HSPs) divided by total genome length; formula d4, sum of all identities found in HSPs divided by overall HSP length; formula d6, sum of all identities found in HSPs divided by total genome length.

Subject strain	dDDH (d0, %)	C.I. (d0, in %)	dDDH (d4, %)	C.I. (d4, in %)	dDDH (d6, %)	C.I. (d6, in %)	G+C content difference (%)
Pseudarthrobacter albicanus NJ-Z5	31,3	[27.9 - 34.9]	27,1	[24.7 - 29.5]	29,2	[26.3 - 32.3]	0,06
Arthrobacter oryzae DSM 25586	36	[32.7 - 39.6]	25,9	[23.6 - 28.4]	32,5	[29.6 - 35.6]	0,04
Pseudarthrobacter psychrotolerans YJ56	22,7	[19.4 - 26.3]	23,4	[21.2 - 25.9]	21,8	[19.0 - 24.8]	2,4
Arthrobacter pascens DSM 20545	24,6	[21.3 - 28.2]	23,4	[21.1 - 25.9]	23,3	[20.5 - 26.4]	1,45
Pseudarthrobacter humi RMG13	23,9	[20.6 - 27.5]	23,2	[20.9 - 25.7]	22,7	[19.9 - 25.8]	2,06
Arthrobacter globiformis NBRC 12137	23	[19.7 - 26.6]	23,2	[20.9 - 25.7]	22	[19.2 - 25.1]	0,93
Pseudarthrobacter sulfonivorans ALL	22,6	[19.3 - 26.2]	23,1	[20.8 - 25.6]	21,7	[18.9 - 24.7]	2,03
Pseudarthrobacter siccitolerans 4J27	22	[18.8 - 25.7]	22,8	[20.5 - 25.2]	21,2	[18.4 - 24.2]	2,01
Arthrobacter cavernae PO-11	22,8	[19.5 - 26.4]	22,8	[20.6 - 25.3]	21,7	[19.0 - 24.8]	0,97
Pseudarthrobacter scleromae CGMCC 1.3601	22,6	[19.3 - 26.2]	22,7	[20.4 - 25.1]	21,6	[18.8 - 24.7]	1,16
Arthrobacter nitrophenolicus SJConT	22,7	[19.4 - 26.3]	22,7	[20.5 - 25.2]	21,7	[18.9 - 24.7]	0,91
Pseudarthrobacter polychromogenes CGMCC 1.1927	22,2	[18.9 - 25.8]	22,7	[20.4 - 25.1]	21,3	[18.5 - 24.3]	1,33
Arthrobacter nitrophenolicus DSM 23165	22,9	[19.6 - 26.5]	22,6	[20.4 - 25.1]	21,8	[19.0 - 24.9]	0,9
Pseudarthrobacter phenanthrenivorans Sphe3	21,9	[18.6 - 25.5]	22,6	[20.4 - 25.1]	21	[18.3 - 24.1]	1,78
Pseudarthrobacter enclensis NIO-1008	22,7	[19.4 - 26.4]	22,3	[20.0 - 24.7]	21,6	[18.8 - 24.7]	0,05

Figure S1. Whole-genome sequence-based GBDP tree. Tree inferred with FastME 2.1.6.1 from GBDP distances calculated from genome sequences. The branch lengths are scaled in terms of GBDP distance formula d5. The numbers above branches are GBDP pseudo-bootstrap support values > 60 % from 100 replications, with an average branch support of 96.3 %. The tree was rooted at the midpoint.

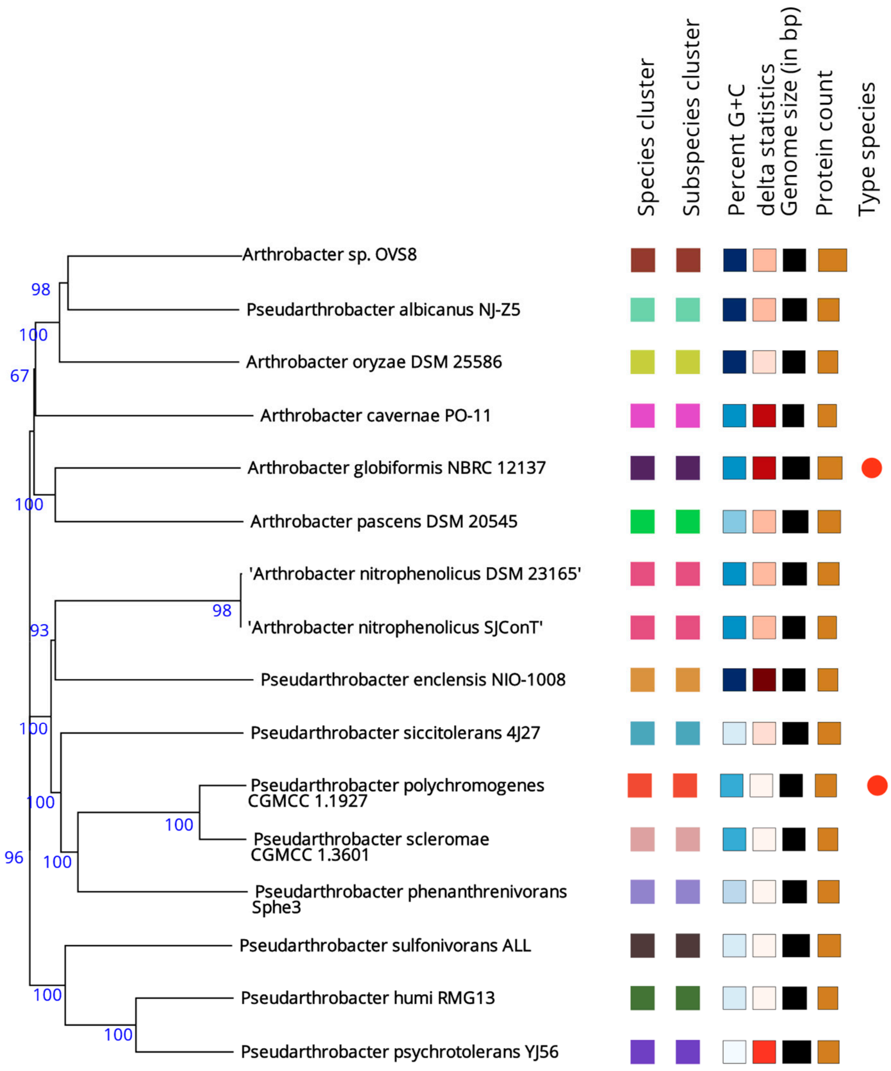

Figure S2. 16S rDNA gene sequence-based GBDP tree. Tree inferred with FastME 2.1.6.1 from GBDP distances calculated from 16S rDNA gene sequences. The branch lengths are scaled in terms of GBDP distance formula d5. The numbers above branches are GBDP pseudo-bootstrap support values > 60 % from 100 replications, with an average branch support of 55.8 %. The tree was rooted at the midpoint.

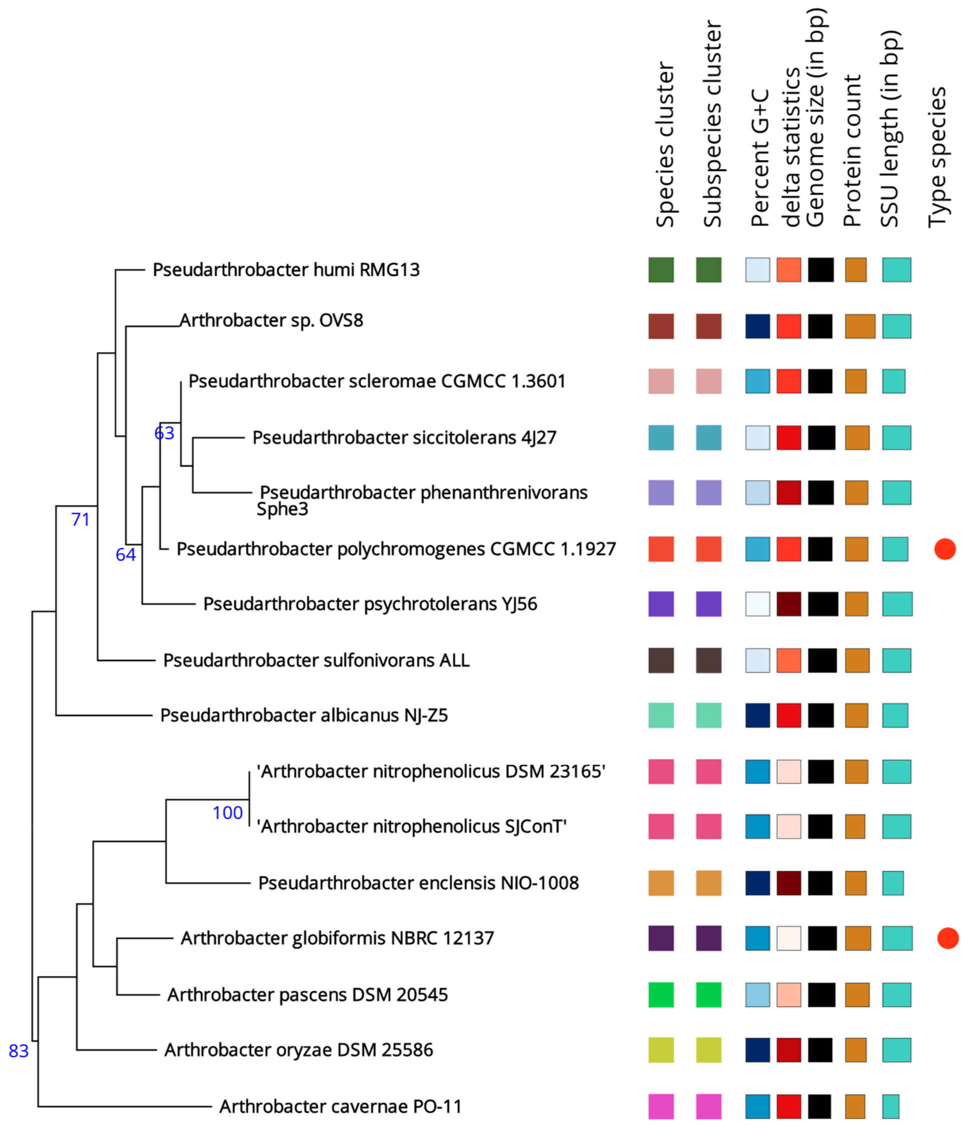

Supplement: Supplementary file 1 [file ijms-24-04845-s001.zip › ijms-2230444-supplementary.pdf]
